# Relationships between physical activity, body mass index, waist circumference and handgrip strength amongst adults from the North West province, South Africa: The PURE study

**DOI:** 10.4102/phcfm.v14i1.3206

**Published:** 2022-05-23

**Authors:** Sindisiwe Shozi, Makama A. Monyeki, Sarah J. Moss, Cindy Pienaar

**Affiliations:** 1 Physical Activity, Sport, and Recreation Research Focus Area, Faculty of Health Sciences, North-West University, Potchefstroom, South Africa; 2 Department of Sport Studies, Faculty of Applied Sciences, Durban University of Technology, Durban, South Africa

**Keywords:** physical activity, handgrip strength, obesity, body mass index, adults, South African

## Abstract

**Background:**

Handgrip strength (HGS) serves as a proxy for the functional ability and its association with body composition (BC) and physical activity (PA) in South African adults are less clear.

**Aim:**

We investigated the relationships between PA, body composition and HGS amongst adults.

**Setting:**

Rural and urban population from North West Province, South Africa.

**Methods:**

A cross-sectional study design was performed on 688 (198 men; 490 women) adults aged 35–70 years from the 2015 measurement wave of the Prospective Urban and Rural Epidemiological (PURE) study from the North West province of South Africa. The International Physical Activity Questionnaire-Short Form (IPAQ-SF) assessed and a dynamometer determined HGS in kilogram. Body mass index (BMI) and waist circumference were used as measures of body composition. Spearman correlation coefficients determined the relationship between PA, BMI and HGS.

**Results:**

In the study, 22% and 26%, respectively, were overweight and obese with women being more overweight and obese compared to men. Sixty percent of the participants met the recommended 150 min or more of moderate to vigorous PA (MVPA) per week. Handgrip strength of the participants in the study was weaker than the published norms. Handgrip strength significantly (*p* < 0.05) differed by age. A significant positive association was found between HGS and BMI. Age negatively (*r* = –0.12; *p* = 0.001) correlated with MVPA per week.

**Conclusion:**

High prevalence of overweight and obesity exists in the current adults’ sample. It was also evident that poor upper limb muscle strength and MVPA were negatively associated with ageing. Given the health implications of poor strength indicators, PA intervention programmes, comprised of strength activities, for an adult population are urgently recommended.

## Background

Previous publications reveal significant positive associations between handgrip strength (HGS), body mass index (BMI) and physical activity in adult men and women,^1,2^ whilst a negative association was observed between HGS and waist circumference.^2^ In addition, a loss in muscle mass is significantly associated with ageing.^3,4,5^ Worldwide, 31.1% of adults are reported to be physically inactive.^6^ Physical inactivity is linked to non-communicable diseases (NCDs) such as Type 2 diabetes mellitus and cancers.^6,7^ Some studies and reports^8,9,10^ indicate that 20% of the global populace within the age range of 18–64 years comply with the minimum physical activity guidelines for improved health.^11^

Studies have also indicated that obese individuals suffer from an early onset of NCDs that is often carried into adulthood.^12,13,14^ Overweight and obesity are identified as a constant negative influence on physical activity, regardless of age.^14,15^ Therefore, the prevention of obesity through regular physical activity should be encouraged as early as possible and should be maintained, to prevent spontaneous tracking into adulthood.^16^ Waist circumference is most often used as the surrogate measure of abdominal or visceral adiposity in clinical and public health settings when working with obesity or overweight individuals.^17^ Kob et al.^18^ reported that muscle architecture which is physical arrangement of muscle fibre at the macroscopic level that determines a muscle’s mechanical function (e.g. infiltration of intramuscular fat), are related to lower physical performance and function. A study by Benavides-Rodriguez et al.^19^ reported a partial mediation of anthropometric parameters (body mass, body mass index and waist circumference) in an association between HGS and muscle mass. Elevated BMI, waist to height ratio (WHtR) and waist circumference (WC) measures are found to be associated with all-cause mortality, diabetes mellitus, cardiovascular morbidity and general mortality.^20,21,22,23,24^

Moderate to vigorous daily physical activity of at least 150 min a week is reported to be associated with increased muscle strength and indicative of one’s quality of life.^25^ Handgrip strength is a proxy measurement tool for measuring the maximum isometric strength of the hand and forearm muscles, as it has demonstrated to be a superior outcome predictor in healthy and ill individuals.^26^ Poor muscle strength, therefore, plays a significant role in reducing the odds of physical and functional limitations with age and more so in obese elderly.^27^ Conversely, a low HGS is associated with being overweight, having excessive body fat and having a high BMI.^28^ Therefore, physical activity is essential in improving muscle strength and decreasing excessive body weight. When it comes to HGS and BMI, an improvement in muscular strength and functional status can be achieved by regular physical activity, which plays a similar role in reducing the odds of being overweight or obese.^27,29^ Regardless of age, good muscular strength and a low BMI can be indicators of better health and good early life nutrition, whereas poor muscle strength and a higher BMI are viewed as associated risk factors for diseases and subclinical diseases.^30^

Available findings on the relationships between HGS, body composition and physical activity also show the effect of gender, with men often outperforming women in HGS.^3^ More physically active women are often classified with a lower BMI score than less physically active women, whilst low levels of physical activity are an indication of extreme vulnerability to continuous weight gain^14^ when women are compared to men.^29.30,31^ Cois and Day,^12^ identified that obesity is associated with advancing age in African women and in South Africa.^12^ Physical activity, muscle mass and strength decrease with age and these decreases lead to sarcopenia (a loss in skeletal muscle mass) associated with a resultant loss in mobility.^12^ Regular participation in physical activity can delay the development of sarcopenia by improving and maintaining muscle mass and strength.

In spite of available information regarding the relationships between physical activity, HGS and body composition, paucity on these relationships exist for adults in African populations, especially from the North West Province of South Africa. The majority of the population in the North West Province is amongst the very poor of the country, reporting multiple health challenges.^32^ A better understanding of the relationships between HGS, body composition and physical activity can motivate intervention programmes to improve health and longevity and minimise the risks associated with physical inactivity. The measurement of HGS as a predictor of functional limitations, functional decline and mortality in older adults is extremely useful and economically viable. Therefore, the purpose of this study was to determine the relationships between physical activity, BMI, WC and HGS amongst adults from the North West province of South Africa.

## Methods

### Study design

The study followed a cross-sectional study design analysing data collected from a total of 688 (men, *n* = 198 and women, *n* = 490) participants from the 2015 measurement wave of the Prospective Urban and Rural Epidemiological (PURE) study conducted in the North West Province of South Afria.^33,34^

Included were native black African participants who had completed data for all outcome variables of height, body mass, BMI, WC and HGS.

### Study population and sampling

The study sample comprised black African adults from both urban and rural areas between the ages of 35–70 years from the 2015 data collected in the North West PURE multidisciplinary international study that started in 2005. For the purpose of this study and taking into consideration the published age-related norms (Table 1) for HGS,^5^ the participants were categorised according to three age groups, namely 42–49, 50–59 and 60–70 years. The data used in this study complied with the inclusion and exclusion criteria of the PURE study^33,34^ according to which, participants were included if they were older or equal to 35 years of age, ≤ to 70 years of age, men and women and ‘apparently healthy’ without any risk factors for NCDs, tuberculosis (TB) or human immunodeficiency virus (HIV), not using chronic medication and eligible participants had to reside in the household. However, participants’ data as it was received from principal investigator (PI) were excluded for analysis if an individual was younger than 35 years of age, older than 70 years of age, using a chronic medication, presented with a temperature above 37 °C and using chronic medication with existing chronic conditions. The detailed methods and population characteristics of the PURE study are not included in this article as they are published elsewhere.^33,34^ Briefly, the design of the PURE study was based on selected countries to achieve substantial socio-economic heterogeneity. For reasons of feasibility, the PURE study undertook comparable sampling from all countries worldwide or from regions within countries. At the start of the study, selected countries were categorised according to high-income (Canada, Sweden and United Arab Emirates), upper-middle-income (Argentina, Brazil, Chile, Malaysia, Poland, South Africa and Turkey), lower middle-income (China, Colombia and Iran) and low-income (Bangladesh, India, Pakistan and Zimbabwe) countries. The categories were further grouped based on country-income levels with a similar number of participants. Participants from high-income and upper-middle-income countries were grouped together as participants from high-income countries.^33,34^ The method of approaching households differed amongst countries but was designed to avoid biases based on levels of risk factors or the prevalence of diseases. Households could participate in the study if at least one member of the household was between the ages of 35–70 years and the household members intended to continue living at their current address for another 4 years. Only individuals who provided written informed consent were enrolled.

**TABLE 1:** Handgrip strength norms.

Age	Handgrip strength norms					
	HGS norms for men			HGS norms for women		
	Poor	Normal	Strong	Poor	Normal	Strong
35–39	< 35.8	35.8–55.6	> 55.6	< 20.3	20.3–34.1	> 34.1
40–44	< 35.5	35.5–55.3	> 55.3	< 18.9	18.9–32.7	> 32.7
45–49	< 34.7	34.7–54.5	> 54.5	< 18.6	18.6–32.4	> 32.4
50–54	< 32.9	32.9–50.7	> 50.7	< 18.1	18.1–31.9	> 31.9
55–59	< 30.7	30.7–48.5	> 48.5	< 17.7	17.7–31.5	> 31.5
60–64	< 30.2	30.2–48.0	> 48.0	< 17.2	17.2–31.0	> 31.0
65–69	< 28.2	28.2–44.0	> 44.0	< 15.4	15.4–27.2	> 27.2
70–99	< 21.3	21.3–35.1	> 35.1	< 14.7	14.7–24.5	> 24.5

*Source*: Adapted from Ramlagan S, Peltzer K, Phaswana-Mafuya N. Hand grip strength and associated factors in non-institutionalised men and women 50 years and older in South Africa. BMC Central Res Notes. 2014;8:1–7. https://doi.org/10.1186/1756-0500-7-8

HGS, handgrip strength.

### Data collection

#### Anthropometrics and body composition

Anthropometric measurements of height, body mass and WC were obtained according to standard procedures described by the International Standard of Advancement of Kinanthropometry (ISAK).^35^ Body mass was measured to the nearest 0.1 kg of body mass by using a precision health scale manufactured by A and D Company of Tokyo, Japan. Stature (height) measurements were recorded to the nearest 0.1 cm using a calibrated stadiometer. Body mass index was calculated from body mass in kilogram divided by height in metre squared (kg/m^2^). Body mass index was categorised according to the American College of Sport Medicine (ACSM) cut points. Body mass index < 18 kg/m² = underweight; 18.5 4 kg/m² – 24.4 kg/m² = normal; BMI 25 4 kg/m² – 29.9 kg/m² = overweight and BMI ≥ 30 kg/m² = obese. Waist circumference measurements were recorded to the nearest 0.1 cm by using a non-stretchable standard Lufkin tape measure manufactured by Cooper Tools of Apex, North Carolina, United States. The WC risk measurements were categorised according to very low (WC < 70 cm in women and < 80 cm in men), low (WC 70 cm – 89 cm in women and 80 cm – 99 cm in men), high (WC 90 cm – 109 cm in women and 100 cm – 120 cm in men) and very high (WC > 110 cm women and WC > 120 cm in men).

#### Handgrip strength

Handgrip strength was measured using a hand-held model (T.K.K.54010 Takei) dynamometer with the participants in a seated position with the elbow of the dominant hand to be tested flexed at a 90º degree angle. The dominant hand was tested twice and both values were recorded in kilogram. Following the procedures prescribed by the World Health Organization, the given steps were followed to take the HGS measurements^9^:

- Set the dynamometer to zero (0).
- Check the fit of the dynamometer to the hand of the participant.
- Adjust by turning the handle to move it up or down, so that the bar rests on the phalanx bone of the index and ring finger.
- Ask the participant to use his or her dominant hand to grab the two pieces of metal, keeping the upper arm close to his or her body and holding his or her forearm at a 90º degree angle to the upper arm.
- When ready, ask the participant to squeeze the dynamometer as hard as he or she can for 3 s.
- Read the dial at eye level and record strength in kilograms to the nearest kilogram.
- Record ‘00’ whenever an attempt was not made.
- Set the dynamometer to zero (0) and repeat the test with the dominant hand.
- Repeat the procedure.

The highest score for HGS in kilograms was used in the data analysis. Data from this study were compared with the HGS norms presented in Table 1.

#### Physical activity

Physical activity was determined with the International Physical Activity Questionnaire – Short Form (IPAQ-SF).^25^ The questionnaire was completed by means of interviews. The participants were requested to report on their time, frequency and duration of physical activity in terms of minutes per day of participation in vigorous and moderate-intensity activities and walking in bouts of at least 10 min during the past seven days. For the purpose of this study, physical activity was expressed in terms of minutes per week spent in vigorous physical activity, moderate physical activity, walking, moderate to vigorous physical activity (MVPA), sedentary activities and the total of physical activity. Physical activity was reported as metabolic equivalent task (MET) minutes/week.^36^

### Data analysis

All the analyses were done using the Statistical Page for Social Sciences (SPSS) version 26 statistical software (IBM SPSS, 26). Normality distribution of the data was determined with the Kolmogorov–Smirnov test. Descriptive statistics were employed to determine the characteristics of the participants and to report the mean, minimum, maximum and standard deviations. Frequency distributions were calculated in percentages for physical activity, BMI, WC and HGS. An independent t-test for parametric and non-parametric variables was calculated to determine gender differences. The chi-square was used to calculate the differences between the categorical variables of physical activity, BMI, WC and HGS. An analysis of variance (ANOVA) was used to determine significant differences between BMI, WC and physical activity (PA) per age group. In order to determine correlations between physical activity, body composition and HGS amongst the participants, Spearman correlation coefficients (*r*) were employed for the total group and for men and women separately. The level of significance was set at *p* ≤ 0.05.

### Ethical considerations

Ethical clearance to conduct this study was obtained from the Health Research Ethics Committee for Humans of the Faculty of Health Sciences at the North-West University (NWU) (ethics number: NWU– 00016-10-A1), and the study conformed to the ethical principles outlined in the Declaration of Helsinki (revised 2004). Anonymised data as received from the principal investigator (PI) was used in the analyses for this manuscript.

## Results

Maximum HGS was observed in middle life (40–49 years) and the age group (50–59 years) with a decrease in the 60–70-year group in both the men and women (Tables 2a and 2b). The HGS performance of the underweight men and women was very low, with a significant increase (*p* < 0.05) in performance in both the normal and overweight group whilst a decrease in HGS was observed in the obese group. Waist circumference significantly differed in the three age categories. In terms of physical activity, both men and women who participated in 150 min or more MVPA per week showed a significant (*p* = 0.002) higher mean HGS compared to the other two physical activity groups. The mean values of the men were high compared to the women across the different physical activity levels.

**TABLE 2a:** Mean handgrip strength in men and women in the different age groups, physical activity levels, body mass index and waist circumference.

Variable	Handgrip strength (kg)							
	Men					Women		
	*N*	Mean ± s.d.	*p*	Mean	s.d.	*N*	Mean ± s.d.	*p*
**Age group**			0.0001					0.0001
40–49 years	34	34.81 ± 11.13		-	-	88	26.29 ± 7.72	
50–59 years	88	34.05 ± 9.20		-	-	235	25.12 ± 6.67	
60–70 years	76	30.28 ± 8.69		-	-	167	23.28 ± 6.45	
**BMI**			0.03					0.0001
Underweight (BMI < 18.5 kg/m²)	57	30.22 ± 8.81		-	-	40	20.8550 ± 5.62	
Normal (BMI = 18.5 kg/m² – 24.4 kg/m²)	100	32.89 ± 8.68		-	-	161	23.9820 ± 5.95	
Overweight (BMI = 25 kg/m² – 29.9 kg/m²)	25	36.41 ± 11.37		-	-	128	25.8859 ± 7.68	
Obese (BMI ≥ 30 kg/m²)	16	34.96 ± 12.05		-	-	161	25.4375 ± 6.96	
**Waiste circumference**			0.096					0.001
WC risk category very low WC < 70 cm (< 28.5 cm) in women and < 80 cm in men	108	-		31.46	8.06	136	22.42 ± 5.86	
WC risk category low WC 70 cm – 89 cm in women and 80 cm – 99 cm in men	73	-		34.58	10.40	180	24.54 ± 6.91	
WC risk category high 90 cm – 109 cm in women and 100 cm – 120 cm in men	17	-		32.88	12.99	138	26.63 ± 6.87	
WC risk category very high > 110 cm women and > 120 cm in men	-	-		-	-	36	26.75 ± 7.67	
**Moderate to vigorous physical activity**			0.003					0.002
Inactive: less than 30 min/week of MVPA	33	28.35 ± 9.73		-	-	54	22.64 ± 7.84	
Insufficiently active: 30–149 min/week of MVPA	33	31.16 ± 8.87		-	-	134	23.64 ± 6.48	
Sufficiently active: 150 min/week or more of MVPA	132	34.22 ± 9.53		-	-	302	25.54 ± 6.87	

BMI, body mass index; WC, waist circumference; MVPA, moderate to vigorous physical activity per week.

**TABLE 2b:** Characteristics according to age groups.

Variables	*N*	Mean	s.d.	Minimum	Maximum	*p*-value*
**Age (year)**						
42–49 years	122	47.45	1.67	42.49	49.99	< 0.001
50–59 years	323	55.03	2.91	50.01	59.96	
60–70 years	243	64.96	3.02	60.00	70.99	
Total	688	57.19	6.93	42.49	70.99	
**Body weight (kg)**						
42–49 years	122	63.25	16.15	30.60	109.90	0.43
50–59 years	323	63.73	16.73	32.50	106.50	
60–70 years	243	65.25	15.84	33.70	105.80	
Total	688	64.19	16.31	30.60	109.90	
**Height (cm)**						
42–49 years	122	159.14	8.32	143.20	177.10	0.95
50–59 years	323	159.40	11.64	15.00	185.80	
60–70 years	243	159.17	7.39	140.70	179.10	
Total	688	159.27	9.75	15.00	185.80	
**BMI (kg/m^2^)**						
42–49 years	122	25.02	6.32	14.53	37.79	0.33
50–59 years	323	25.03	6.66	11.47	39.86	
60–70 years	243	25.80	6.28	14.81	39.93	
Total	688	25.30	6.47	11.47	39.93	
**WC (cm)**						
42–49 years	122	85.89	14.18	55.30	122.80	0.13
50–59 years	323	86.33	14.84	55.20	122.60	
60–70 years	243	88.49	13.424	59.10	116.60	
Total	688	87.01	14.26	55.20	122.80	
**Vigmin/wk**						
42–49 years	122	88.03	226.10	0.00	1800	0.002
50–59 years	323	62.12	129.37	0.00	900	
60–70 years	243	34.96	94.02	0.00	900	
Total	688	57.12	142.54	0.00	1800	
**Modmin/wk**						
42–49 years	122	295.16	275.85	0.00	1260	0.03
50–59 years	323	322.37	321.08	0.00	1260	
60–70 years	243	255.19	265.71	0.00	1260	
Total	688	293.82	295.81	0.00	1260	
**Walkmin/wk**						
42–49 years	122	253.93	247.42	20.00	1680	0.02
50–59 years	323	282.32	290.23	0.00	3360	
60–70 years	243	220.82	224.78	0.00	1680	
Total	688	255.57	262.35	0.00	3360	
**MVPA (min/wk)**						
42–49 years	122	383.19	433.49	0.00	2700.00	0.007
50–59 years	323	384.49	392.62	0.00	1860.00	
60–70 years	243	290.14	304.95	0.00	1800.00	
Total	688	350.94	374.34	0.00	2700.00	
**HGS (kg)**						
42–49 years	122	28.66	9.56	6.00	66.20	0.001
50–59 years	323	27.55	8.43	9.10	61.90	
60–70 years	243	25.46	7.91	5.77	48.50	
Total	688	27.01	8.54	5.77	66.20	

* , *p*-value differences between the groups.

BMI, body mass index; WC, waist circumference; Vigmin/wk, vigorous intensity exercise in minutes per week; Modmin/wk, moderate intensity exercise in minutes per week; walkmin/wk, walking in minutes per week; MVPA, moderate to vigorous physical activity per week and HGS, handgrip strength.

The results showed that 29% of men and women were underweight, 22% were overweight, and 26% were obese (Tables 3a and 3b). In terms of gender, the results showed that women were significantly (*p* < 0.001) more overweight (33%) and obese (26%) compared to men. The WC of 28% of women (*p* = 0.001) was found to be within the higher risk category (90 cm – 109 cm) compared to only 8% of that of men. The percentage of women who were classified as having a very high WC risk (> 110 cm) is 5% higher compared to that of men. Men were not reported to be included within the very high WC risk category (> 120 cm). Men significantly (*p* = 0.001) reported more physical activity than women, whilst more of women (27%) were categorised with insufficient active levels of 30–149 min/week of MVPA than men (17%). The same percentage (17%) was reported for inactive men and for men who were classified under an insufficient level of MVPA, whilst the percentage of women with regard to the physical activity categories varied.

**TABLE 3a:** The percentage scores of the participants with regard to body mass index, waist circumference and physical activity categories for the total group, men, women and per age group.

Variables	Total group		Men		Women		*p*-value of the differences
	Freq.	%	Freq.	%	Freq.	%	
**Age groups**							
42–49 years	122	18	34	17	88	18	< 0.001
50–59 years	323	47	88	44	235	48	
60–70 years	243	35	76	38	167	34	
Total	688	100	198	100	490	100	
**BMI categories**							
Underweight	97	14	57	29	40	8	
Normal	261	38	100	50	161	33	< 0.001
Overweight	153	22	25	13	128	26	
Obese	177	26	16	8	161	33	
Total	688	100	198	100	490	100	
**WC Categories**							
WC risk category very low if WC is < 70 cm (< 28.5 cm) in women and < 80 cm in men	244	36	108	55	136	28	< 0.001
WC risk category low 70–89 cm (28.5 cm – 35.0 cm) in women and 80–99 cm in men	253	37	73	37	180	37	
WC risk category high 90 cm – 109 cm (35.5 cm – 43.0 cm) in women and 100 cm – 120 cm in men	155	22	17	9	138	28	
WC risk category very high if WC is > 110 (> 43.5) women and > 120 cm in men	36	5	-	-	36	7	
Total	688	100	198	100	490	100	
**PA Categories**							
Inactive: less than 30 min/week of MVPA	87	13	33	17	54	11	< 0.001
Insufficiently active: 30–149 min/week of MVPA	167	24	33	17	134	27	
Sufficiently active: 150 min/week or more of MVPA	434	63	132	66	302	62	
Total	688	100	198	-	490	100	

BMI, body mass index; WC, waist circumference; PA, physical activity; MVPA, moderate to vigorous physical activity per week.

**TABLE 3b:** The percentage scores of the participants with regard to body mass index, waist circumference and physical activity according to age groups.

Variables	Age 42–49		50–59		60–70		*p*-value of the differences
	Freq.	%	Freq.	%	Freq.	%	
**BMI categories**							
Underweight	19	16	55	17	23	9	< 0.001
Normal	50	41	117	36	94	39	
Overweight	19	16	70	22	64	26	
Obese	34	28	81	25	62	26	
Total	122	100	323	100	243	100	
**WC categories**							
WC risk category very low < 70 cm in women and < 80 cm in men	46	38	124	38	74	30	< 0.001
WC risk category low 70 cm – 89 cm in women and 80 cm – 99 cm in men	47	39	116	36	90	37	
WC risk category high 90 cm – 109 cm in women and 100 cm – 120 cm in men	25	20	63	20	67	28	
WC risk category very high > 110 cm women and > 120 cm in men	4	3	20	6	12	5	
Total	122	100	323	100	243	100	
**PA categories**							
Inactive: less than 30 min/week of MVPA	10	8	38	12	39	16	< 0.001
Insufficiently active: 30–149 min/week of MVPA	30	25	74	23	63	26	
Sufficiently active: 150 min/week or more MVPA	82	67	211	65	141	58	
Total	122	100	323	100	243	100	

Freq, frequency; BMI, body mass index; WC, waist circumference; PA, physical activity; MVPA, moderate-vigorous physical activity.

Underweight was significantly high in the age groups 42–49 years and 50–59 years, respectively, whilst overweight and obesity significantly (*p* < 0.001) varied in all the age groups (Table 3a, 3b). In the WC categories, the results showed that both the 42–49 year and 50–59 year age groups had similar percentages (20%) of high WC risk categories of 90 cm – 109 cm in women and 100 cm – 120 cm in men (*p* < 0.001). The risk of being associated with a very high WC risk category was lower in the three different age groups, namely, 42–49 years (3%), 50–59 years (6%) and 60–70 years (5%).

The mean ages were significantly (*p <* 0.001) different with regard to each of the age groups (age group 42–49 years; mean: 47.46 ± 1.67 years; age group 50–59 years, mean: 55.04 ± 3.91 years and age group 60–70 years, mean: 64.96 ± 3.02 years) (Table 4). The middle-age group significantly performed better in vigorous (*p* = 0.002) and moderate activities (*p* = 0.03) and walked more minutes per week (*p* = 0.03) compared to the 60–70-year-old group. In terms of the total MVPA, the middle-age groups performed significantly (*p* = 0.007) better than the age group 60–70 years. The mean for HGS significantly (*p* < 0.001) decreases with increased age in all three of the age groups (42–49 years, mean: 28.67 ± 9.56 kg; 50–59 years, mean: 27.55 ± 8.43 kg; 60–70 years, mean: 25.47 ± 7.9 kg).

**TABLE 4:** Mean, standard deviation (±s.d.) and *p*-value of the differences with regard to body mass index, waist circumference, physical activity and handgrip strength for men and women, according to the different age groups.

Variables	Age group 42–49 years			Age group 50–59 years			Age group 60–70 years		
	*N*	Mean ± s.d.	*p*-value of gender differences	*N*	Mean ± s.d.	*p*-value of gender differences	*N*	Mean ± s.d.	*p*-value of gender differences
**Age (year)**									
Men	34	47.42 ± 1.76	0.88	88	55.18 ± 2.92	0.58	76	64.91 ± 3.00	0.94
Women	88	47.47 ± 1.65		235	54.98 ± 2.92		167	64.98 ± 3.04	
**Body weight (kg)**									
Men	34	61.55 ± 13.99	0.47	88	57.44 ± 12.29	< 0.001	76	60.51 ± 14.72	< 0.001
Women	88	63.91 ± 16.94		235	66.09 ± 17.57		167	67.42 ± 15.90	
**Height (cm)**									
Men	34	167.21 ± 5.48	< 0.001	88	167.37 ± 8.12	< 0.001	76	164.97 ± 6.51	< 0.001
Women	88	156.02 ± 7.05		235	156.42 ± 11.37		167	156.53 ± 6.18	
**BMI (kg/m^2^)**									
Men	34	22.03 ± 4.99	0.001	88	20.48 ± 4.22	< 0.001	76	22.16 ± 5.14	< 0.001
Women	88	26.18 ± 6.42		235	26.74 ± 6.62		167	27.46 ± 6.06	
**WC (cm)**									
Men	34	81.37 ± 13.06	0.03	88	78.69 ± 10.67	< 0.001	76	83.20 ± 12.96	< 0.001
Women	88	87.63 ± 14.28		235	89.19 ± 15.18		167	90.89 ± 12.97	
**Vigmin/wk**									
Men	34	180.74 ± 328.69	0.002	88	101.82 ± 154.87	< 0.001	76	49.87 ± 122.57	0.04
Women	88	52.22 ± 144.42		235	47.26 ± 115.33		167	28.17 ± 77.15	
**Modmin/wk**									
Men	34	382.06 ± 328.49	0.01	88	437.39 ± 386.53	0.02	76	307.17 ± 300.06	0.13
Women	88	261.59 ± 246.60		235	279.30 ± 281.91		167	231.53 ± 245.85	
**Walkmin/wk**									
Men	34	346.47 ± 366.69	0.61	88	327.50 ± 270.66	0.06	76	239.14 ± 256.21	0.69
Women	88	218.18 ± 171.76		235	265.40 ± 296.01		167	212.49 ± 209.24	
**MVPA (min/week)**									
Men	34	562.79 ± 592.28	0.07	88	539.20 ± 476.11	< 0.005	76	357.04 ± 363.80	0.10
Women	88	313.80 ± 322.90		235	326.55 ± 339.81		167	259.70 ± 269.80	
**HGS (kg)**									
Men	34	34.81 ± 11.13	< 0.001	88	34.05 ± 9.20	< 0.001	76	30.28 ± 8.69	< 0.001
Women	88	26.29 ± 7.72		235	25.12 ± 6.67		167	23.27 ± 6.45	

BMI, body mass index; WC, waist circumference; Vigmin/wk, vigorous intensity exercise in minutes per week; Modmin/wk, moderate intensity exercise in minutes per week; walkmin/wk, walking in minutes per week; MVPA, moderate to vigorous physical activity per week; HGS, handgrip strength.

No significant difference was found in women’s BMI for the three age groups (25 kg/m^2^) (*p* = 0.28), whilst a borderline significant difference (*F* = 2.932, *p* = 0.06) was found in men’s BMI (Table 5). Significant differences (*p* < 0.05) were found in the HGS of both men and women. The 60–70 years’ group performed poorer than the middle-age group. The men in the middle-age group participated significantly more in vigorous activities per week compared to the 60–70 years’ group. A similar trend, although not significant (*p* > 0.05), was found in women.

**TABLE 5:** The descriptive characteristics (mean, s.d., minimum, maximum and *p*-value of the differences between the groups) of men and women.

Variables	Men					Women				
	*N*	Mean	s.d.	*F*	*p*-value of the differences between groups	*N*	Mean	s.d.	*F*	*p*-value of the differences between groups
**Weight (kg)**										
42–49 years	34	61.55	13.99	1.597	0.20	88	63.91	16.94	1.241	0.29
50–59 years	88	57.44	12.29			235	66.09	17.56		
60–70 years	76	60.51	14.73			167	67.42	15.90		
**Height (cm)**										
42–49 years	34	167.21	5.48	2.567	0.08	88	156.02	7.05	.091	0.913
50–59 years	88	167.37	8.12			235	156.42	11.37		
60–70 years	76	164.97	6.51			167	156.53	6.18		
**BMI (kg/m^2^)**										
42–49 years	34	22.03	4.99	2.932	0.06	88	26.18	6.43	1.268	0.28
50–59 years	88	20.48	4.23			235	26.74	6.61		
60–70 years	76	22.16	5.14			167	27.46	6.06		
**WC (cm)**										
42–49 years	34	81.37	13.06	2.907	0.06	88	87.63	14.28	1.602	0.20
50–59 years	88	78.69	10.67			235	89.19	15.18		
60–70 years	76	83.20	12.96			167	90.89	12.97		
**HGS (kg)**										
42–49 years	34	34.81	11.13	4.308	0.01	88	26.29	7.72	6.528	0.002
50–59 years	88	34.05	9.20			235	25.12	6.67		
60–70 years	76	30.28	8.69			167	23.27	6.45		
**Vigmin/wk**										
42–49 years	34	180.74	346.69	5.551	0.01	88	52.22	144.42	1.957	0.14
50–59 years	88	101.82	154.87			235	47.26	115.33		
60–70 years	76	49.87	122.57			167	28.17	77.15		
**Modmin/wk**										
42–49 years	34	382.06	328.49	2.895	0.06	88	261.59	246.60	1.602	0.20
50–59 years	88	437.39	386.53			235	279.30	281.91		
60–70 years	76	307.17	300.06			167	231.53	245.85		
**Walkmin/wk**										
42–49 years	34	346.47	366.68	2.599	0.08	88	218.18	171.76	2.563	0.08
50–59 years	88	327.50	270.66			235	265.40	296.01		
60–70 years	76	239.14	256.20			167	212.49	209.24		
**Sitmin/wk**										
42–49 years	34	235.29	128.52	5.007	0.01	88	275.68	144.78	1.449	0.24
50–59 years	88	275.68	179.51			235	263.66	126.58		
60–70 years	76	337.63	175.79			167	286.77	140.09		
**MVPA/wk**										
42–49 years	34	562.79	592.28	3.978	0.02	88	313.80	332.90	2.261	0.11
50–59 years	88	539.20	476.11			235	326.55	339.81		
60–70 years	76	357.04	363.80			167	259.70	269.80		
Total	198	473.33	466.69			490	301.48	317.19		

BMI, body mass index; WC, waist circumference; Subs, subscapular skinfold; Vigmin/wk, vigorous intensity exercise in minutes per week; Modmin/wk, moderate intensity exercise in minutes per week; walkmin/wk, walking in minutes per week; sitmin/wk= sitting in minutes per week; WHtR, waist to hip ratio; MVPA, moderate to vigorous physical activity per week.

A significant positive correlation was found between hand grip strength with body mass and MVPA. Body mass index related positively with body mass, WC and age, but a negative correlation was found with MVPA (Table 6). Handgrip strength positively correlated with body mass, height and MVPA whilst an inverse correlation was found with age. Moderate to vigorous physical activity had a negative (*r* = –0.12; *p* = 0.001) correlation with age.

**TABLE 6:** Correlation coefficients (*r*) between body mass index, waist circumference, physical activity and handgrip strength of the total group.

Variables	Weight	Height	BMI	Waist	WHtR	MVPA	Age	HGS	Triceps
**Total group**									
Weight (cm)									
*r*	-	0.16**	0.92**	0.90**	0.81**	−0.02	0.04	-	-
*p*	-	0.00	0.00	0.00	0.00	0.63	0.33	-	-
Height (cm)									
*r*	0.16**	-	−0.20**	−0.02	−0.29**	0.06	−0.004	-	-
*p*	0.00	-	0.00	0.58	0.00	0.12	0.92	-	-
BMI (kg/m^2^)									
*r*	0.92**	−0.20**	-	0.91**	0.92**	−0.04	0.04	-	-
*p*	0.00	0.00	-	0.00	0.00	0.35	0.28	-	-
Waist (cm)									
*r*	0.90**	−0.02	0.91**	-	0.95**	−0.07	0.06	-	-
*p*	0.00	0.58	0.00	-	0.00	0.08	0.09	-	-
HGS (kg)									
*r*	0.16**	0.44**	−0.01	0.06	−0.06	0.25**	−0.15**	-	-
*p*	0.00	0.00	0.82	0.09	0.12	< 0.001	< 0.001	-	-
MVPA (min/wk)									
*r*	−0.02	0.06	−0.04	−0.07	−0.07	-	−0.12**	-	-
*p*	0.63	0.12	0.35	0.08	0.06	-	0.001	-	-
Age (year)									
*r*	0.04	−0.004	0.04	0.06	0.06	−0.12**	-	-	-
*p*	0.33	0.92	0.28	0.09	0.10	0.001	-	-	-
**Men**									
Weight (kg)									
*r*	-	0.28**	0.92**	0.90**	0.79**	−0.03	0.03	0.278**	-
*p*	-	0.000	0.000	0.000	0.000	0.61	0.62	0.000	-
Height (cm)									
*r*	0.28**	-	−0.06	0.11	−0.17*	−0.14	−0.13	0.184**	-
*p*	0.000	-	0.37	0.11	0.02	0.05	0.07	0.009	-
BMI (kg/m^2^)									
*r*	0.92**	−0.06	-	0.89**	0.90**	0.008	0.07	0.240**	-
*p*	0.000	0.37	-	0.000	0.000	0.91	0.32	0.001	-
WC (cm)									
*r*	0.90**	0.11	0.89**	-	0.95**	−0.07	0.12	0.195**	-
*p*	0.000	0.11	0.000	-	0.000	0.29	0.08	0.006	-
HGS (kg)									
*r*	0.28**	0.18**	0.24**	0.19**	0.15*	0.24**	−0.23**	-	-
*p*	0.000	0.01	0.001	0.01	0.04	0.001	0.001	-	-
MVPA (min/wk)									
*r*	−0.03	−0.14	0.01	−0.07	−0.04	-	−0.19**	0.243**	-
*p*	0.61	0.05	0.91	0.29	0.57	-	0.006	0.001	-
Age (year)									
*r*	0.03	−0.13	0.07	0.12	0.16*	−0.19**	-	−0.233**	-
*p*	0.62	0.07	0.32	0.08	0.02	0.006	-	0.001	-
**Women**									
Weight (kg)									
*r*	-	0.34**	0.95**	0.90**	0.82**	0.03	0.04	0.28**	0.76**
*p*	-	0.000	0.000	0.000	0.000	0.43	0.33	0.000	0.000
Height (cm)									
*r*	0.34**	-	0.05	0.19**	−0.06	0.03	0.01	0.35**	0.13**
*p*	0.000	-	0.27	0.000	0.16	0.49	0.80	0.000	0.005
BMI (kg/m^2^)									
*r*	0.95**	0.05	-	0.89**	0.89**	0.03	0.05	0.18**	0.77**
*p*	0.000	0.27	-	0.000	0.000	0.51	0.29	0.000	0.000
WC (cm)									
*r*	0.90**	0.19**	0.89**	-	0.96**	−0.008	0.06	0.23**	0.72**
*p*	0.000	0.000	0.000	-	0.000	0.86	0.18	0.000	0.000
HGS (kg)									
*r*	0.28**	0.35**	0.18**	0.23**	0.14**	0.18**	−0.18**	-	0.22**
*p*	0.000	0.000	0.000	0.000	0.002	0.000	0.000	-	0.000
MVPA (min/wk)									
*r*	0.03	0.03	0.03	−0.008	−0.01	-	−0.11*	0.18**	−0.05
*p*	0.43	0.49	0.51	0.86	0.75	-	0.01	0.000	0.25
Age (year)									
*r*	0.04	0.01	0.05	0.06	0.06	−0.11*	-	−0.18**	−0.007
*p*	0.33	0.80	0.29	0.18	0.15	0.01	-	0.000	0.87

BMI, body mass index; WC, waist circumference; WHtR, waist-to-hip ratio; MVPA, moderate to vigorous physical activity per week; HGS, handgrip strength.

* , A correlation is significant at the 0.05 level (two tailed).

** , A correlation is significant at the 0.01 level (2-tailed).

When the data were analysed separately for men and women, the measurements of men differed significantly with regard to body mass, WC and BMI, compared to the measurements of women. Significant positive correlations were found between body mass, WC and BMI in both men and women, whilst a negative relationship was found between BMI and height (*r* = –0.6; *p* = 0.37) in men. A positive relationship was found between all of the anthropometric measures of height, body mass, WC and BMI. However, an inverse correlation was found between handgrip strength and age (*r* = –0.23; *p* = 0.001) in men and (*r* = –0.18, *p* = 0.001) in women. Furthermore, handgrip strength was significantly (*p* < 0.05) and positively correlated with MVPA.

A positive, significant (*p* = 0.000) correlation was found between four of the five anthropometric measurements. Handgrip strength correlated positively with the body composition measurements whilst a negative correlation was found between HGS (*r* = –0.18; *p* = 0.000) and age. In addition, MVPA negatively correlated with age, respectively, in men (*r* = –0.18, *p* = 0.01) and in women (*r* = –0.11; *p* = 0.01).

## Discussion

The purpose of this study was to determine the relationships between physical activity, anthropometric measures of height, body mass, BMI, WC and HGS amongst adults from the North West Province in South Africa. The results showed a significant negative relationship between HGS with MVPA and age. The results of the study are congruent with the findings of Smith et al.^37^ who found that maximum strength is observed in middle life (40–49 years) and between the ages of 50–59 years. Compared to the middle-age group, men and women aged 60–70 years presented decreases in HGS, which is associated with poor functional hand performance.

Overweight and obesity classifications were 22% and 26%, of the total sample. Women were more overweight (26%) and obese (33%) compared to men (13% overweight and 8% obese). The findings of this study are similar to previous studies^38,39,40,41,42^ with the prevalence of overweight and obesity consistently high in most countries (i.e. The United States, Spain, United Kingdom, Mexico, Australia and Ghana) exceeding 30% in the elderly age group for both men and women. A survey conducted by Vorster et al.^43^ in transitional African communities in the North West Province of South Africa, also reported that inactivity – independent of the degree of urbanisation – is significantly (*p* = 0.0007) associated with high obesity levels that are similar to the findings of this study.

Furthermore, the study reported a negative correlation between age and MVPA, but a significant decline in physical activity with increased age, especially in the elderly (60–70 year age group), was found. This negative correlation in the study between MVPA and age can be because the participants were mostly physically inactive, reporting a sedentary lifestyle that contributes to limited functionality. Also, the age group of individuals in our study has been reported by other studies to be more prone to various health problems (NCDs, such as high blood pressure), which could have impacted their ability to readily take part in unsupervised physical activity compared to other age groups (i.e. children and adolescents).^2,10,44^ Other studies^44,45^ have reported a similar decline in MVPA with increased age, resulting in functional limitations that can indicate their health status and predict future events, such as disabilities and various illnesses.

The study also reported a negative correlation between age and HGS. However, a poor HGS performance was constantly associated with increased age (elderly individuals around the age of 60 years). The negative correlation can, therefore, be because of HGS decrease with an increase in age in which hand muscles consisting of small muscles deteriorate faster than the larger muscle groups during ageing, as the target age group was found to be prevalent with the common health-related risk factors of advancing age. Robert et al.^46^ reported that the precision of HGS measurements can be influenced by protocol, such as allowance for hand size, hand dominance, posture, joint position, effort and encouragement, frequency of testing and time of the day – regardless of age. An explanation for the negative correlation between HGS and age may be because of factors, such as hand size, joint posture and time of the measurements that can influence a correlation between HGS and age. Moreover, the findings of the study found no correlation between HGS and anthropometric measurements, such as BMI and WC for the total group measured. However, when the data were further analysed according to gender, a positive correlation between HGS and all of the anthropometric measurements was noted for both men and women. These results are similar to the findings reported in a study where a weaker HGS was associated with being overweight, having a high body fat percentage and high BMI values, whilst a stronger HGS was associated with individuals who reported a normal BMI value.^26^

An association between physical activity and HGS was noted in this study in both older men and women, illustrating that physical activity and HGS significantly decrease with age. These findings are consistent with other studies.^1,33,47^ Body mass index values increased with age and women consistently reported higher BMI values and poor physical activity performance compared to the values of men. These findings may imply that improved physical activity can benefit individuals with weak muscles and physical activity can be used to influence the quality of life.^45,48^ Other studies^27,30,49,50^ explained the importance of physical activity in muscle strength and BMI based on their role in supporting muscle mass by decelerating the progression of physical functional limitations and disability in older adults. Therefore, physical activity remains a key element in preventing and/or managing NCDs and disabilities compromised by poor muscle strength.^50,2,51,52^

The study had several strengths and limitations that should be taken into consideration when the results of the study are interpreted. The study utilised a large sample and included data from both urban and rural areas in the North West Province. In spite of these strengths, given the study’s cross-sectional nature, causal relationships could not be determined. The bias and recall problems linked to the use of subjective questionnaire-based assessment of physical activity can be considered a limitation of this study. Future studies should therefore incorporate objective measures of physical activity. Additionally, the lack of racial distribution data of the particapants was a limitation of the study when considering some physiological and socio-economic differences, which might occur in relation to the observed findings. Other study limitations of the study were that fat-free mass was not measured and the participants’ socio-economic status was not considered. The socio-economic status of the participants can influence physical activity participation, BMI, WC and muscle strength. The findings of the study are limited to the North West Province and therefore cannot be generalised.

## Conclusion

A high BMI is associated with less MVPA per week, especially in women compared to men. Furthermore, the results showed that ageing significantly affects functional performance concerning HGS and MVPA in both men and women. Urgent strategic and supervised physical activity intervention programmes for older men and women are needed to ensure optimal functional performance and independent living.
